# A quantitative account of genomic island acquisitions in prokaryotes

**DOI:** 10.1186/1471-2164-12-427

**Published:** 2011-08-24

**Authors:** Tom E Roos, Mark WJ van Passel

**Affiliations:** 1 Genomics Coordination Center, University Medical Center Groningen, University of Groningen, Groningen, The Netherlands; 2 Netherlands Bioinformatics Centre, Nijmegen, The Netherlands; 3 Systems and Synthetic Biology, Wageningen University, Wageningen, the Netherlands

**Keywords:** Genomic Islands, genome signature, horizontal gene transfer

## Abstract

**Background:**

Microbial genomes do not merely evolve through the slow accumulation of mutations, but also, and often more dramatically, by taking up new DNA in a process called horizontal gene transfer. These innovation leaps in the acquisition of new traits can take place via the introgression of single genes, but also through the acquisition of large gene clusters, which are termed Genomic Islands. Since only a small proportion of all the DNA diversity has been sequenced, it can be hard to find the appropriate donors for acquired genes via sequence alignments from databases. In contrast, relative oligonucleotide frequencies represent a remarkably stable genomic signature in prokaryotes, which facilitates compositional comparisons as an alignment-free alternative for phylogenetic relatedness.

In this project, we test whether Genomic Islands identified in individual bacterial genomes have a similar genomic signature, in terms of relative dinucleotide frequencies, and can therefore be expected to originate from a common donor species.

**Results:**

When multiple Genomic Islands are present within a single genome, we find that up to 28% of these are compositionally very similar to each other, indicative of frequent recurring acquisitions from the same donor to the same acceptor.

**Conclusions:**

This represents the first quantitative assessment of common directional transfer events in prokaryotic evolutionary history. We suggest that many of the resident Genomic Islands per prokaryotic genome originated from the same source, which may have implications with respect to their regulatory interactions, and for the elucidation of the common origins of these acquired gene clusters.

## Background

The advent of whole genome sequencing has drastically altered our perspective on life's evolutionary history. Bacterial genomes are now known to be largely mosaics made up of horizontally transferred genes [1-4]. In fact, many bacteria that cause disease, like those that cause plague, meningitis, tetanus or cholera have only become virulent after they acquired virulence genes [5-8], highlighting the impact of horizontal gene transfer on human health [9]. In many cases, questions remain about the natural reservoir of these acquired genes [10,11].

These horizontally transferred genes are not necessarily acquired one at a time. Numerous bacterial genomes show clusters of recently acquired genes that are known as Genomic Islands (GIs) [3,12-16]. Even though many GIs have unknown functions, some of these acquired gene clusters are involved in pathogenicity (the Pathogenicity Associated Islands), though several other clustered collective functions are known (metabolic islands, degradation islands *et cetera*) [17,18]. We hypothesise that, when several GIs reside in a single genome, it is possible that a single donor has been responsible for multiple gene transfer events to that host.

In a previous study, we found that compositionally similar sequences can be clustered together, and a genomic acquisition account of large acquired gene clusters can be established [19]. Such alignment-free compositional analyses focus on the similarity between two sequences with respect to their relative dinucleotide frequencies. In brief, relative dinucleotide frequencies are known to be typical for a given genome, a genomic signature, and similar between related species. This parameter can be used to assess the similarity in composition between different sequences, for example in binning sequences that are thought to be derived from the same organism [20-24]. Compositional analyses have been used before to detect compositionally anomalous genes [25], which could subsequently be identified as putative horizontally acquired genes. In addition, similar comparative analyses have been applied to metagenomic datasets, in which genomic fragments were assigned to their probable host based on their compositional similarities [26,27]. However, few attempts have been made to compositionally compare clusters of acquired genes, in order to indicate common donors, analogous to assigning donors to sequences from metagenomic libraries.

Here we focus on the acquisition accounts of GIs that are identified in the genomes of a large collection of bacterial and archaeal species [28]. By comparing the compositional similarities of all GIs that reside in the same genome, for a large number of genomes, conservative estimates of the maximum number of compositionally distinct donors can be assessed. This will shed new light onto the evolutionary histories of prokaryotes, and the quantitative dynamics of recurrent horizontal gene transfer events of large gene clusters.

## Methods

Analyses were carried out as described previously [19], with a few modifications for scaling up the analyses. In brief, GIs were obtained from IslandViewer [28] at http://www.pathogenomics.sfu.ca/islandviewer/download.php, selecting only those species/genera having genome sizes > 800 kb and the GIs with sizes > 10 kb. Smaller genomes are thought to be mostly devoid of GIs since they often represent intracellular symbionts, whereas the 10 kb cut-off is based on previous publications concerning sizes of GIs [29]. IslandViewer is a computational tool that integrates different genomic island prediction software suits; two sequence composition prediction methods (IslandPick [30], SIGI-HMM [31]) and a comparative GI prediction method (IslandPath-DIMOB [13]). Regions that are identified with IslandViewer are annotated as putative genomic islands, and included in our GI set [28]. This database may not cover all large acquired gene clusters, but does allow for large-scale compositional analyses.

With this collection of GIs, the compositional relatedness of each GI was subsequently compared with its respective genome. This was done by comparing the composition of the GI, with the compositions of all genomic fragments of the same size [25,32]. Next, all GIs residing in the same genome were compared with each other by calculating the average dinucleotide relative abundance difference, or genomic dissimilarity (δ*) [20], after which we cluster all compositionally similar GIs per genome, based on their genomic dissimilarity values using δρ-Web and Compare_Islands [19,25], respectively. More information on these methods can be found at the website http://deltarho.amc.nl. In brief, distance matrices of GI comparisons per genome revealed the GIs that have a lower genomic dissimilarity than a conservative threshold sequence to its host genome. With respect to these conservative thresholds of relatedness, we included in each comparison a chromosomal fragment of 15 kb with a very low relative dissimilarity with its genome. The relative dissimilarity signifies the dissimilarity between a query sequence and the rest of the genome; the dissimilarity of the query is relative to the collection of non-overlapping genomic fragments of identical size as the query. The relative dissimilarity is expressed as a percentage of genomic fragments with a lower genomic dissimilarity than the query sequence. A relative dissimilarity of 95% signifies that 95% of all non-overlapping genomic fragments of identical size as the query is more similar to the genome than the query is. The threshold sequences are based on progressively lower relative dissimilarity values. Core Islands CI-25, CI-10, CI-5 and CI-0 represent the four threshold sequences with relative dissimilarity values of 25%, 10%, 5% and 0%. In other words, a Core Island from a specific genome is compositionally very similar to its host. In order to test that different GIs from the same genome originate from a same donor species, the GIs need to be compositionally more similar to each other than the Core Island is to its host genome. Thus, if GIs meet these similarity thresholds, we score these GIs as clusters that have a compositionally similar background, and therefore likely a common origin. In some instances, compositionally similar GIs are not clustered together due to a high compositional similarity between a GI from a predicted cluster with a GI outside of that specific cluster. The similarity threshold between the unclustered GI and one of the other GIs in the cluster is not met. These problems in an unambiguous interpretation of the clustering are categorized as 'conflicts', and subsequently all GIs from that genome are excluded from the cluster analyses in order to reduce potential misclassifications. An example of an analysis with a clustering conflict is given in Additional File 1, which gives the compositional distance matrix of six GIs from the genome of *Clostridium botulinum *Ba4_657 (NC_012658), relative to the CI-25 threshold sequence. These conflicts are removed from the analyses in an attempt to obtain a conservative dataset with few ambiguities.

A set of stand-alone scripts is available from the authors (at https://trac.nbic.nl/brsp200901_vanpassel/wiki), with both instructions on how to perform the analyses for GI sets automatically as well as the raw data for the analyses presented here. It iterates all calculations for each applicable GI-host and GI-GI combination within a host, allowing the user to choose different cut-off values of compositional dissimilarity (i.e., the Core Islands), as well as GI size. For individual GI/genome comparisons, Compare_Islands can be used at http://deltarho.amc.nl [19].

In order to test to test the accuracy of our clustering approach and cut-offs, we simulate a clustering fidelity by analyzing how frequently fragments from the same genome are clustered together when a pool of phylogenetically unrelated 15 kb sequences are compared. For each of the four thresholds, 100 analyses are carried out, each consisting of a set of 100 sequences; 90 originating from distinct genera, and ten non-biological randomized sequences. The 90 sequences originate from 30 genomes, with three fragments per genome, and two out of these three have a relative dissimilarity like the threshold that is being simulated. The third sequence has a relative dissimilarity of 50% with its host genome. In this simulation, the accuracy of the clustering is expressed as a percentage, which indicates how often the threshold sequences are clustered with sequences from the same host genome.

## Results

**Figure 1:**
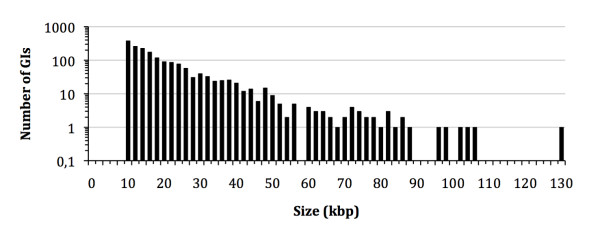
**Size distribution of 1787 Genomic Islands > 10 kb in 246 genome sequences (note the logarithmic scale on the vertical axis)**. The GIs are binned per 2 kb in size.

**Figure 2:**
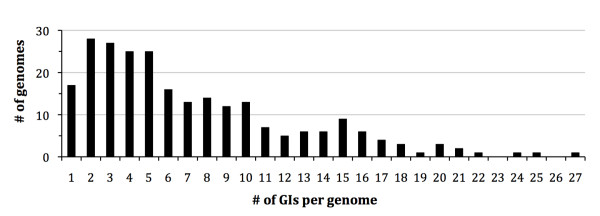
**Number of Genomic Islands per genome for the 246 genomes tested (Genome size > 800 kb, with GIs size > 10 kb and no conflicts)**.

First, we extracted all Genomic Islands from IslandViewer (December 2009, [28]), amounting to a total of 5447 sequences between 2.2 and 143 kb in size, originating from 339 distinct genomes. After applying the conservative criteria discussed in the Material and Methods section (genome size > 800 kb, GI size > 10 kb, monochromosomal genomes, no internal conflicts in the clustering approach using the CI-10 cut-off threshold sequence), we maintained 1787 GIs (33%) that vary in size from 10 kb to 130 kb (average 20.7 kb, Figure 1). These GIs are present in 246 genome sequences, which represent 88 species in 45 genera (Additional File 2). This means on average 7 GIs per genome, varying from 1 (in 17 genomes) to 27 (in *Xanthomonas oryzae *MAFF 311018) GIs per genome (Figure 2). These 1787 GIs, using the compositional threshold of CI-10, were used in our subsequent investigations, unless noted otherwise.

**Figure 3:**
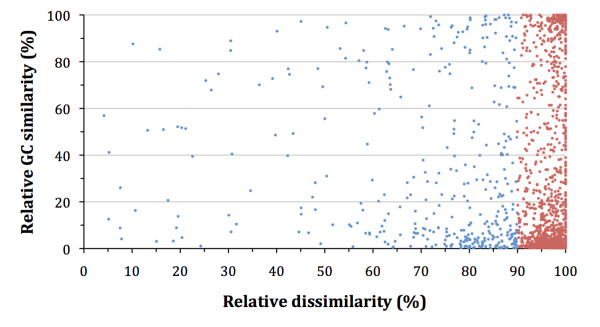
**Distribution of the relative compositional similarity and GC similarity of all GIs (1787) with their respective genomes, with 1395 (78%, in red) of the GIs having a relative dissimilarity of 90%**.

Second, we analyzed the composition dissimilarities of these GIs with their respective host chromosomes similar to previous analyses on comparisons between plasmids and host chromosomes [33]. Of the 1787 GIs, 1394 (78%) are compositionally anomalous compared to their host genome (with a genomic dissimilarity score higher than that of 90% of the genomic fragments of equal length, Figure 3). Of these 1394 GIs, a large number (683 GIs, 49%) have a very low GC content compared to fragments of identical length from their respective host genomes (i.e., lower than 95% of identical sized fragments from their respective genome). Out of the 1787 GIs, only 11 GIs (0.6%) have a lower genomic dissimilarity with the host genome than with the threshold sequence CI-10, meaning that these GIs are compositionally extremely similar to their respective host genomes (Additional File 2).

**Figure 4:**
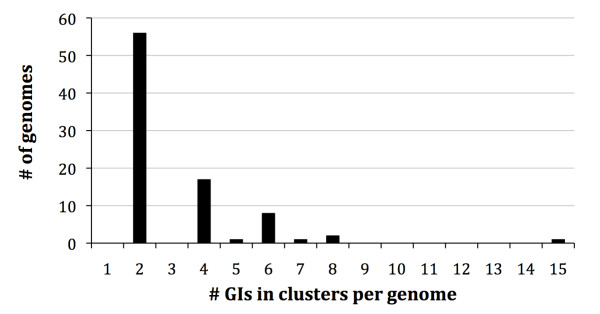
**Number of clustered GIs per genome**.

**Figure 5:**
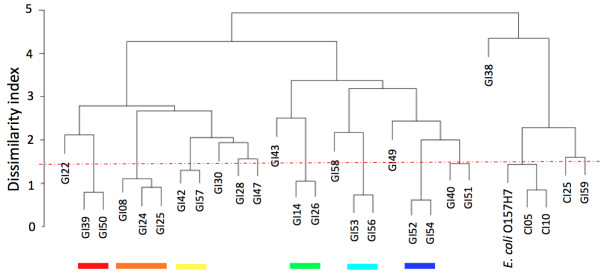
**Clustering of the 24 Genomic Islands > 10 kb in *Escherichia coli *O157H7 strain Sakai in seven clusters and nine singletons**. Below the cut-off value (red line; dissimilarity < 1.44, see Additional File 2), seven clusters are identified (six clusters with two GIs, and one with three GIs), with a total of 15 Genomic Islands (indicated with seven colored bars). The GIs and their numbers are identified in Additional File 5.

Third, we tested all GIs per genome for their compositional dissimilarity. In 86 genomes (of 44 species in 28 genera) we identify 134 clusters, including a total of 271 GIs (15.3% of the 1770 GIs that reside in genomes with at least 2 GIs, Figure 4, Additional File 3). The number of clustered GIs per genome varies between the minimal 2 GIs in a single cluster (in 56 genomes), to 15 GIs in a total of 7 clusters (in the EHEC strain *E. coli *O157H7 Sakai, Figure 5). The only clusters that contain three GIs occur in three genomes *Bradyrhizobium *ORS278, *Escherichia coli *O157H7 strain Sakai and *Rhodobacter sphaeroides *ATCC 17025. In *Bradyrhizobium *ORS278, the three GIs that are clustered together show a high compositional similarity to the host genome sequence. Two of these in fact belong to the 11 GIs that are compositionally very similar to their respective genomes, and therefore these two GIs are unlikely to represent horizontal transfer events. For *E. coli *O157H7 strain Sakai and *R. sphaeroides *ATCC 17025, we tested whether the three clustered GIs are more similar than a set of five sequences belong to the 10% most compositionally similar sequences of the genome (Tables 1 and 2, respectively). We find that for both *E. coli *O157H7 strain Sakai and *R. sphaeroides *ATCC 17025, the GIs that are clustered together in threes, are on average equally or more similar to each other than the five sequences that represent the conservative genome signature (Tables 1 and 2).

**Table 1:** Compositional comparison of Core Islands e1-e5 (with relative dissimilarities of 10%) of *Escherichia coli *O157H7 with each other (underlined), and Genomic Islands with each other (bold)

				Genomic dissimilarity values (δ*)								
	Start coordinate	End coordinate	size (bp)	*E. coli *	e1	e2	e3	e4	e5	GI8	GI24	GI25
***E. coli ***	0	5498450	5498450	0	18,6	18,5	18,5	18,7	18,9	47,2	58,1	49,0
e1	4770000	4785000	15001	18,6	0	22,3	24,7	29,2	30,2	59,8	72,7	65,8
e2	2445000	2460000	15001	18,5	22,3	0	16,2	22,5	28,0	58,3	68,6	63,2
e3	1695000	1710000	15001	18,5	24,7	16,2	0	12,2	21,6	53,0	60,8	57,7
e4	135000	150000	15001	18,7	29,2	22,5	12,2	0	29,7	59,4	67,2	61,5
e5	1455000	1470000	15001	18,9	30,2	28,0	21,6	29,7	0	49,8	54,7	47,0
**GI8**	892240	903808	11568	47,2	59,8	58,3	53,0	59,4	49,8	**0**	**24,4**	**24,9**
**GI24**	2924490	2936721	12231	58,1	72,7	68,6	60,8	67,2	54,7	**24,4**	**0**	**22,0**
**GI25**	3193144	3204209	11065	49,0	65,8	63,2	57,7	61,5	47,0	**24,9**	**22,0**	**0**

**Table 2:** Compositional comparison of Core Islands r1-r5 (with relative dissimilarities of 10%) of *Rhodobacter sphaeroides *with each other (underlined), and Genomic Islands with each other (bold)

				Genomic dissimilarity values (δ*)								
	Start coordinate	End coordinate	size (bp)	*R. sphaeroides *	r1	r2	r3	r4	r5	GI1	GI3	GI4
***R. sphaeroides ***	0	3217726	3217726	0	16,3	16,9	16,8	16,8	16,9	42,1	40,6	44,0
r1	2400000	2415000	15001	16,3	0	25,9	26,2	22,1	29,9	41,7	43,7	45,9
r2	990000	1005000	15001	16,9	25,9	0	31,0	31,1	23,8	48,1	46,2	47,1
r3	1620000	1635000	15001	16,8	26,2	31,0	0	25,8	20,3	41,8	46,4	51,1
r4	2910000	2925000	15001	16,8	22,1	31,1	25,8	0	21,0	29,6	33,1	33,5
r5	2310000	2325000	15001	16,9	29,9	23,8	20,3	21,0	0	43,8	40,2	40,0
**GI1**	2883355	2905795	22440	42,1	41,7	48,1	41,8	29,6	43,8	**0**	**16,9**	**18,9**
**GI3**	2085503	2112636	27133	40,6	43,7	46,2	46,4	33,1	40,2	**16,9**	**0**	**12,8**
**GI4**	1575936	1597159	21223	44,0	45,9	47,1	51,1	33,5	40,0	**18,9**	**12,8**	**0**

In genomes with multiple chromosomes, we test for GIs that are compositionally very similar to each other, yet reside on a different replicon. Out of 110 GIs identified in this set of genomes, 38 GIs are assigned to a total of 19 clusters (Additional File 4). Out of these 38 GIs, ten (26%), all of them in *Burkholderia *genomes, are not located on the same chromosome.

When relaxing the similarity threshold by using the genome signature difference between the Core Island 25 (CI-25) and the genome, we observe only 1370 GIs in a total of 220 genome sequences that meet our criteria. With this more lenient threshold, a total of 16 GIs are now compositionally more similar to the host genome than the cut-off sequence CI-25 is to the genome. A total of 383 GIs (out of 1353 GIs that reside with at least one other GI in a genome; 28%) are now grouped together in 185 clusters, with 13 clusters containing three GIs.

In contrast, when making the composition similarity threshold substantially more conservative (i.e., using CI-5), we find 2047 GIs in a total of 260 genome sequences. Only 9 GIs are now compositionally more similar to the host genome when compared to the cut-off sequence CI-5. Still, there are 99 clusters containing 202 GIs (out of 2030 GIs that reside with at least one other GI in a genome; 10%), which show very high compositional similarity within each cluster (Table 3). Finally, using threshold CI-0, in which GIs need to be compositionally more similar to each other than the genomic fragment that has a nearly identical dinucleotide composition as the host genome, we find only 40 GIs that form 20 clusters. These 40 GIs represent only 1.8% of the total number of GIs included in this analysis.

**Table 3:** Overview of the characteristics of the GI analyses using decreasing similarity thresholds (for all GIs > 10 kb)

	Stringency	Total number of GIs	Number of genomes	GI < CI	Clusters	GIs in clusters*	Percentage clustered (%)	Prediction Accuracy (%)
CI-0	++++	2191	267	1	20**	40**	1.8	99.9
CI-5	+++	2047	260	9	99	202	10.0	98.6
CI-10	++	1787	246	11	134	271	15.3	97.5
CI-25	+	1370	220	16	185	383	28.3	94.8
					
Total analyzed		2609	322					

The totals represent the total numbers in the original data set from IslandViewer.

*) The percentage of clustered GIs (second last column) excludes 17 GIs from the total number of GIs (third column), since there are 17 genomes with a single GI only, and with less than two GIs there can be no clustering.

**) Six out of 20 clusters contain in fact largely identical Genomic Islands, which explains their high compositional similarity.

Finally, in order to validate the accuracy of the clustered GIs per threshold, we simulated cluster assignments by comparing sets of 100 15 kb fragments from random prokaryotic genomes for their compositionally most similar fragment. Each set of 100 fragments consists of three fragments per species, for 30 species of distinct genera, and includes 10 random synthetic sequences with no biological significance. For each threshold, the simulation was carried out 100 times. Accuracy is expressed as the percentage in which a sequence is found to be most similar to another sequence from the same genome. These values are used as proxies for the correct assignment of a sequence from the same genome, and range from 99.9% accurate for the strictest threshold of CI-0, to 94.8% accurate for the CI-25 threshold (Table 3).

## Discussion

By comparing Genomic Islands from 339 bacterial and archaeal chromosomes, we explore the dynamics of the genome-specific acquisition accounts on a large scale. These analyses show us that in numerous cases, distinct GIs in particular genomes are remarkably similar in composition. This leads us to speculate that, using a conservative similarity cut-off, in 15.3% of the cases, multiple acquisition events of GIs have taken place from a donor with a very similar base composition as the acceptor.

For this goal, we developed a suite of scripts that allows users to customize these analyses by modifying the minimal GI length, or the similarity cut-off sequence (i.e., the genomic Core Island of each genome with a certain genomic compositional dissimilarity). By increasing the stringency to the very conservative CI-5, which means that GIs need to be more similar to each other in composition than 95% of the genomic fragments, we still find that 10% of the tested GIs can be grouped together to a total of 99 GI clusters. However, we investigate the accuracy of our assessments by simulating the clustering efficiency in a randomized sample of sequences with different thresholds. We find that the prediction accuracy according to this simulation is > 94,8% even for the least conservative compositional threshold (CI-25). This gives credibility to our findings of substantial recurrent transfer events from the same donor to the same host. In *Bradyrhizobium *ORS278, we find a cluster containing three GIs, two of which cannot be considered compositionally dissimilar from the genome. In this case, these GIs may have been residing for a substantial amount of time in the genome, and have ameliorated to the host's genome composition [34,35].

This approach does not discriminate between separate introgressions of multiple compositionally very similar sequences from a common donor, and the post-acquisition intragenomic dispersal of a large Genomic Island. Technically, this is of little importance, since in both cases the host of the distinct GIs would be a similar donor. With respect to compositionally similar GIs that reside on separate chromosomes, we find that that incoming GIs seem to be indiscriminate between the replicon it integrates in, or that subsequent dispersal throughout the genome can result in a move to a different replicon.

The association of GIs with virulence factors [36] emphasize the significant role of acquired gene clusters in the evolution of numerous pathogens. Investigations into the repetitive acquisition of GIs from a common source may help identifying potential donors of these sequences through for example the association with species-specific sequence motifs such as DNA uptake sequences [37]. Also, a common origin of compositionally similar clusters may result in common regulatory modules, interactions or mobilizing capacities. For example, a study into small regulatory RNAs (sRNAs) on Genomic Islands in *Salmonella typhimurium *revealed that sRNAs mainly affect the expression of flanking genes [38]. If Genomic Islands disperse throughout the genome, our analysis would facilitate the identification of potential associated regulatory targets that are no longer adjacent.

Unfortunately, the forces that shape the genome signatures of prokaryotes are still unknown. It has been speculated that they could include species-specific properties such as DNA modifications, replication and repair mechanisms [20], though recently statistical support has been found for an environmental influence on the oligonucleotide compositions [39], which could mean that a similar environment could also cause similarities in genome signature. For compositional comparisons such as described here, it is of interest to understand what conditions shape the composition of DNA to which extent, in order to pinpoint potential pitfalls in grouping Genomic Islands.

## Conclusions

Even when lacking sequence alignments, numerous large acquired gene clusters in sequenced genomes can be associated with each other individually via substantial compositional similarities. Our analysis suggests, backed up by simulations, that in many cases recurring horizontal gene transfer events have taken place between a donor and acceptor organism. These analyses do not only quantify these events, but also enable further investigations into the origin of these Genomic Islands, and even help analyzing possible interactions between related sequences.

## Authors' contributions

MWJvP conceived the study, participated in the design of the software, analyzed the data and wrote the manuscript. TER designed the software, analyzed the data and helped draft the manuscript. Both authors read and approved the final manuscript.

## Supplementary Material

Additional file 1 **Example of a GI clustering conflict in *Clostridium botulinum *Ba4 657**. Example of a GI clustering conflict. In *Clostridium botulinum *Ba4 657, six GIs larger than 10 kb are identified by IslandViewer. The CI-25 threshold sequence has a genomic dissimilarity to its genome of 30,86. GI-1 and GI-5 are compositionally more similar to each other (δ* of 22,7), as are GI-2 and GI-5 (δ* of 27,7). However, GI-1 and GI-2 are much more dissimilar (δ* of 42,1), and therefore could be considered as a clustering conflict. Click here for file

Additional file 2 **Complete table for all genomes that contain GIs**. Complete table for all genomes (> 800 kb) that contain GIs ( > 10 kb), and have no conflicts in the genome. In green are highlighted the cases where clusters of three GIs are found. The dissimilarity cut-off is expressed in the genomic dissimilarity value between the Core Island (in this case, CI-10) and the genome sequence. Click here for file

Additional file 3 **List of genomes with clustered GIs**. List of genomes with clusters in which more than 1 GI are located, using the compositional threshold of CI-10. Click here for file

Additional file 4 **Clustered GIs in multichromosomal genomes**. Clusters of GIs in genomes with multiple chromosomes using cut-off of CI-10. Highlighted in green are clustered GIs that are located on a different replicon. Click here for file

Additional file 5 **Characteristics of all Genomic Islands in this study**. Characteristics of all Genomic Islands (5447) analyzed in this study, including their number in the genome of occurrence. The 1787 GIs that comply with the criteria (Genome > 800 kb, GI size > 10 kb, no conflicts in the clustering analysis and using cut-off threshold CI-10) are separated from the rest of the GIs by a blank line. Click here for file
