# Supplementary material for: A quantitative account of genomic island acquisitions in prokaryotes
Source: BMC Genomics. 2011 Aug 24;12:427. doi: 10.1186/1471-2164-12-427 (PMC3176501; doi:10.1186/1471-2164-12-427)
Supplement: Additional file 1 — Example of a GI clustering conflict in Clostridium botulinum Ba4 657. Example of a GI clustering conflict. In Clostridium botulinum Ba4 657, six GIs larger than 10 kb are identified by IslandViewer. The CI-25 threshold sequence has a genomic dissimilarity to its genome of 30,86. GI-1 and GI-5 are compositionally more similar to each other (δ* of 22,7), as are GI-2 and GI-5 (δ* of 27,7). However, GI-1 and GI-2 are much more dissimilar (δ* of 42,1), and therefore could be considered as a clustering conflict. [file 1471-2164-12-427-S1.DOCX]

Supplementary File 1. Example of a GI clustering conflict. In *Clostridium botulinum* Ba4 657, six GIs larger than 10 kbp are identified by IslandViewer. The CI-25 threshold sequence has a genomic dissimilarity to its genome of 30,86. GI-1 and GI-5 are compositionally more similar to each other (δ* of 22,7) then to the CI-25 threshold, as are GI-2 and GI-5 (δ* of 27,7). However, GI-1 and GI-2 are much more dissimilar (δ* of 42,1), and therefore are considered a clustering conflict.

|  |  |  |  | **Genomic dissimilarity values (δ*)** | | | | | | | |
| --- | --- | --- | --- | --- | --- | --- | --- | --- | --- | --- | --- |
|  | **Start coordinate** | **End coordinate** | **size (bp)** | ***Clostridium botulinum* Ba4 657** | **CI-25** | **GI-1** | **GI-2** | **GI-3** | **GI-4** | **GI-5** | **GI-6** |
| *Clostridium botulinum* Ba4 657 | 1 | 3977794 | 3977794 | 0 | 30,86 | 94,76 | 89,67 | 92,12 | 88,85 | 77,67 | 135,47 |
| CI-25 | 225000 | 239999 | 15000 | 30,86 | 0 | 110,1 | 113,7 | 111,7 | 103,5 | 100,9 | 160 |
| GI-1 | 912612 | 925321 | 12709 | 94,76 | 110,1 | 0 | 42,1 | 47,5 | 34,2 | 22,7 | 55,1 |
| GI-2 | 956395 | 972560 | 16165 | 89,67 | 113,7 | 42,1 | 0 | 38,5 | 47,3 | 27,7 | 80,5 |
| GI-3 | 2221400 | 2235394 | 13994 | 92,12 | 111,7 | 47,5 | 38,5 | 0 | 43,8 | 49,2 | 91 |
| GI-4 | 2238624 | 2268970 | 30346 | 88,85 | 103,5 | 34,2 | 47,3 | 43,8 | 0 | 37,2 | 73,7 |
| GI-5 | 3076332 | 3112879 | 36547 | 77,67 | 100,9 | 22,7 | 27,7 | 49,2 | 37,2 | 0 | 65,7 |
| GI-6 | 3669398 | 3693917 | 24519 | 135,47 | 160 | 55,1 | 80,5 | 91 | 73,7 | 65,7 | 0 |
