# Supplementary material for: A quantitative account of genomic island acquisitions in prokaryotes
Source: BMC Genomics. 2011 Aug 24;12:427. doi: 10.1186/1471-2164-12-427 (PMC3176501; doi:10.1186/1471-2164-12-427)
Supplement: Additional file 2 — Complete table for all genomes that contain GIs. Complete table for all genomes (> 800 kb) that contain GIs ( > 10 kb), and have no conflicts in the genome. In green are highlighted the cases where clusters of three GIs are found. The dissimilarity cut-off is expressed in the genomic dissimilarity value between the Core Island (in this case, CI-10) and the genome sequence. [file 1471-2164-12-427-S2.DOCX]

Supplementary File 2. Complete table for all genomes (>800 kbp) that contain GIs (>10 kbp), and have no conflicts in the genome. In green are highlighted the cases where clusters of three GIs are found. The dissimilarity cut-off is expressed in the genomic dissimilarity value between the Core Island (in this case, CI-10) and the genome sequence.

| **Organism** | **Accession** | **Nr of basepairs** | **Nr of GIs longer than threshold** | **Nr of GIs with d* with Genome < cutoff** | **Nr of clusters under cutoff** | **Total nr of GIs in clusters under cutoff** | **Total nr of GIs** | **Dissimilarity Cut-off (using CI-10)** |
| --- | --- | --- | --- | --- | --- | --- | --- | --- |
| *Escherichia coli* O157H7 | NC 002695 | 5577000 | 24 | 0 | 7 | 15 | 59 | 1,44 |
| *Mycobacterium marinum* M | NC 010612 | 6731639 | 21 | 1 | 4 | 8 | 24 | 1,38 |
| *Shewanella baltica* OS155 | NC 009052 | 5200625 | 20 | 0 | 4 | 8 | 30 | 1,11 |
| *Escherichia coli* C ATCC 8739 | NC 010468 | 4814022 | 15 | 0 | 3 | 6 | 27 | 1,26 |
| *Escherichia coli* K 12 substr W3110 | AC 000091 | 4712709 | 17 | 0 | 3 | 6 | 32 | 1,22 |
| *Mycobacterium ulcerans* Agy99 | NC 008611 | 5712058 | 17 | 0 | 3 | 6 | 29 | 1,09 |
| *Pseudomonas putida* F1 | NC 009512 | 6045107 | 17 | 0 | 3 | 6 | 29 | 1,29 |
| *Pseudomonas putida* GB 1 | NC 010322 | 6165265 | 17 | 0 | 3 | 6 | 23 | 1,40 |
| *Rhodobacter sphaeroides* ATCC *17025* | NC 009428 | 3263694 | 13 | 0 | 3 | 7 | 16 | 1,26 |
| *Shewanella* W3-18-1 | NC 008750 | 4775643 | 20 | 1 | 3 | 6 | 26 | 1,09 |
| *Streptococcus pyogenes* SSI-1 | NC 004606 | 1921337 | 10 | 2 | 3 | 6 | 13 | 1,24 |
| *Xanthomonas oryzae* KACC10331 | NC 006834 | 5012031 | 25 | 0 | 3 | 6 | 31 | 1,38 |
| *Sulfolobus islandicus* L S 2 15 | NC 012589 | 2775362 | 8 | 0 | 2 | 4 | 11 | 1,55 |
| *Bifidobacterium longum infantis* ATCC 15697 | NC 011593 | 2873216 | 15 | 0 | 2 | 4 | 42 | 1,41 |
| *Bradyrhizobium* ORS278 | NC 009445 | 7563110 | 18 | 2 | 2 | 5 | 36 | 1,15 |
| *Clostridium botulinum* A2 Kyoto | NC 012563 | 4214640 | 7 | 0 | 2 | 4 | 21 | 1,86 |
| *Clostridium botulinum* F Langeland | NC 009699 | 4052464 | 5 | 0 | 2 | 4 | 20 | 1,81 |
| *Escherichia coli* APEC O1 | NC 008563 | 5154626 | 14 | 0 | 2 | 4 | 51 | 1,30 |
| *Escherichia coli* O157 H7 EC4115 | NC 011353 | 5651677 | 22 | 0 | 2 | 4 | 56 | 1,41 |
| *Methylobacterium extorquens* PA1 | NC 010172 | 5549314 | 9 | 0 | 2 | 4 | 12 | 1,56 |
| *Salmonella enterica* Choleraesuis | NC 006905 | 4823639 | 13 | 0 | 2 | 4 | 40 | 1,21 |
| *Salmonella enterica* serovar Agona SL483 | NC 011149 | 4867213 | 10 | 0 | 2 | 4 | 30 | 1,23 |
| *Salmonella enterica* serovar Dublin CT 02021853 | NC 011205 | 4912093 | 11 | 0 | 2 | 4 | 27 | 1,33 |
| *Shewanella baltica* OS223 | NC 011663 | 5219415 | 11 | 0 | 2 | 4 | 24 | 1,10 |
| *Shewanella halifaxensis* HAW EB4 | NC 010334 | 5301588 | 9 | 0 | 2 | 4 | 19 | 1,32 |
| *Shigella boydii* CDC 3083 94 | NC 010658 | 4681940 | 18 | 0 | 2 | 4 | 44 | 1,22 |
| *Stenotrophomonas maltophilia* K279a | NC 010943 | 4920428 | 11 | 0 | 2 | 4 | 26 | 1,47 |
| *Xanthomonas oryzae* MAFF 311018 | NC 007705 | 5010792 | 27 | 0 | 2 | 4 | 38 | 1,37 |
| *Xylella fastidiosa* | NC 002488 | 2717582 | 12 | 0 | 2 | 4 | 20 | 1,70 |
| *Yersinia pseudotuberculosis* IP32953 | NC 006155 | 4812453 | 13 | 0 | 2 | 4 | 24 | 1,24 |
| *Methanococcus maripaludis* S2 | NC 005791 | 1684868 | 2 | 0 | 1 | 2 | 2 | 1,32 |
| *Sulfolobus islandicus* M 16 27 | NC 012632 | 2730865 | 5 | 0 | 1 | 2 | 8 | 1,48 |
| *Sulfolobus islandicus* Y G 57 14 | NC 012622 | 2740659 | 4 | 0 | 1 | 2 | 12 | 1,51 |
| *Anaeromyxobacter dehalogenans* 2CP-C | NC 007760 | 5085101 | 7 | 0 | 1 | 2 | 20 | 1,43 |
| *Bacillus cereus* AH820 | NC 011773 | 5378436 | 6 | 0 | 1 | 2 | 10 | 1,48 |
| *Bacillus cereus* G9842 | NC 011772 | 5464296 | 7 | 0 | 1 | 2 | 13 | 1,48 |
| *Bordetella bronchiseptica* | NC 002927 | 5415453 | 9 | 0 | 1 | 2 | 24 | 1,35 |
| *Bordetella pertussis* | NC 002929 | 4144564 | 13 | 0 | 1 | 2 | 23 | 1,25 |
| *Clostridium botulinum* A | NC 009495 | 3942444 | 3 | 0 | 1 | 2 | 17 | 1,75 |
| *Clostridium botulinum* A3 Loch Maree | NC 010520 | 4049948 | 5 | 0 | 1 | 2 | 22 | 1,83 |
| *Clostridium botulinum* A ATCC 19397 | NC 009697 | 3918643 | 3 | 0 | 1 | 2 | 16 | 1,65 |
| *Clostridium botulinum* B1 Okra | NC 010516 | 4014780 | 8 | 0 | 1 | 2 | 20 | 1,78 |
| *Clostridium botulinum* Ba4 657 | NC 012658 | 4034620 | 6 | 0 | 1 | 2 | 21 | 1,95 |
| *Escherichia coli* 536 | NC 008253 | 5009476 | 12 | 0 | 1 | 2 | 37 | 1,26 |
| *Escherichia coli* HS | NC 009800 | 4709875 | 12 | 0 | 1 | 2 | 27 | 1,27 |
| *Escherichia coli* IAI39 | NC 011750 | 5205384 | 21 | 0 | 1 | 2 | 49 | 1,30 |
| *Escherichia coli* K 12 substr MG1655 | NC 000913 | 4705957 | 16 | 0 | 1 | 2 | 32 | 1,18 |
| *Escherichia coli* UMN026 | NC 011751 | 5276406 | 14 | 0 | 1 | 2 | 37 | 1,29 |
| *Escherichia coli* UTI89 | NC 007946 | 5138109 | 11 | 0 | 1 | 2 | 46 | 1,29 |
| *Lactobacillus casei* ATCC 334 | NC 008526 | 2936625 | 9 | 0 | 1 | 2 | 14 | 1,15 |
| *Legionella pneumophila* Corby | NC 009494 | 3627563 | 7 | 0 | 1 | 2 | 9 | 1,11 |
| *Listeria innocua* | NC 003212 | 3054226 | 4 | 0 | 1 | 2 | 5 | 1,31 |
| *Listeria monocytogenes* Clip81459 | NC 012488 | 2954300 | 3 | 0 | 1 | 2 | 4 | 1,27 |
| *Methylobacterium extorquens* AM1 | NC 012808 | 5590056 | 8 | 0 | 1 | 2 | 14 | 1,40 |
| *Methylobacterium extorquens* DM4 | NC 012988 | 6028679 | 7 | 1 | 1 | 2 | 10 | 1,54 |
| *Methylobacterium populi* BJ001 | NC 010725 | 5883305 | 14 | 0 | 1 | 2 | 17 | 1,35 |
| *Pseudomonas syringae* pv B728a | NC 007005 | 6180751 | 15 | 0 | 1 | 2 | 37 | 1,20 |
| *Rhizobium leguminosarum* bv viciae 3841 | NC 008380 | 5129387 | 8 | 0 | 1 | 2 | 16 | 1,28 |
| *Rickettsia massiliae* MTU5 | NC 009900 | 1380340 | 4 | 0 | 1 | 2 | 4 | 1,32 |
| *Salmonella enterica* Paratypi ATCC 9150 | NC 006511 | 4650733 | 10 | 0 | 1 | 2 | 27 | 1,30 |
| *Salmonella enterica* serovar Enteritidis P125109 | NC 011294 | 4752789 | 9 | 0 | 1 | 2 | 28 | 1,20 |
| *Salmonella enterica* serovar Gallinarum 287 91 | NC 011274 | 4725250 | 9 | 0 | 1 | 2 | 25 | 1,17 |
| *Salmonella enterica* serovar Newport SL254 | NC 011080 | 4896608 | 8 | 0 | 1 | 2 | 28 | 1,29 |
| *Salmonella enterica* serovar Paratyphi A AKU 12601 | NC 011147 | 4647252 | 10 | 0 | 1 | 2 | 27 | 1,28 |
| *Salmonella enterica* serovar Paratyphi C RKS4594 | NC 012125 | 4902124 | 9 | 0 | 1 | 2 | 32 | 1,22 |
| *Salmonella typhimurium* LT2 | NC 003197 | 4926824 | 14 | 0 | 1 | 2 | 35 | 1,23 |
| *Shewanella* ANA-3 | NC 008577 | 5043236 | 8 | 0 | 1 | 2 | 14 | 1,30 |
| *Shewanella baltica* OS195 | NC 009997 | 5423673 | 14 | 0 | 1 | 2 | 33 | 1,08 |
| *Shewanella* MR-4 | NC 008321 | 4773520 | 9 | 0 | 1 | 2 | 15 | 1,21 |
| *Shewanella* MR-7 | NC 008322 | 4861076 | 8 | 0 | 1 | 2 | 18 | 1,31 |
| *Shewanella pealeana* ATCC 700345 | NC 009901 | 5248504 | 10 | 0 | 1 | 2 | 21 | 1,21 |
| *Shewanella putrefaciens* CN-32 | NC 009438 | 4725781 | 15 | 0 | 1 | 2 | 27 | 1,16 |
| *Shigella boydii* Sb227 | NC 007613 | 4584392 | 19 | 0 | 1 | 2 | 39 | 1,21 |
| *Shigella flexneri* 2a 2457T | NC 004741 | 4665060 | 20 | 0 | 1 | 2 | 41 | 1,28 |
| *Sinorhizobium meliloti* | NC 003047 | 3706337 | 6 | 0 | 1 | 2 | 9 | 1,11 |
| *Staphylococcus aureus aureus* MRSA252 | NC 002952 | 2944085 | 8 | 0 | 1 | 2 | 12 | 1,50 |
| *Stenotrophomonas maltophilia* R551 3 | NC 011071 | 4639312 | 6 | 0 | 1 | 2 | 19 | 1,32 |
| *Streptococcus equi zooepidemicus* | NC 012470 | 2180581 | 7 | 0 | 1 | 2 | 11 | 1,33 |
| *Streptococcus pneumoniae* D39 | NC 008533 | 2075346 | 6 | 0 | 1 | 2 | 14 | 1,46 |
| *Xanthomonas campestris* 8004 | NC 007086 | 5222261 | 14 | 0 | 1 | 2 | 31 | 1,56 |
| *Xanthomonas campestris* ATCC 33913 | NC 003902 | 5148705 | 15 | 0 | 1 | 2 | 32 | 1,54 |
| *Xanthomonas campestris* B100 | NC 010688 | 5151560 | 11 | 0 | 1 | 2 | 23 | 1,49 |
| *Xanthomonas campestris vesicatoria* 85-10 | NC 007508 | 5252445 | 15 | 0 | 1 | 2 | 34 | 1,54 |
| *Xanthomonas citri* | NC 003919 | 5249491 | 13 | 0 | 1 | 2 | 27 | 1,51 |
| *Yersinia pestis* Angola | NC 010159 | 4568601 | 15 | 0 | 1 | 2 | 27 | 1,04 |
| *Yersinia pseudotuberculosis* PB1 | NC 010634 | 4762700 | 10 | 0 | 1 | 2 | 19 | 1,17 |
| *Methanococcus maripaludis* C5 | NC 009135 | 1806201 | 5 | 0 | 0 | 0 | 5 | 1,44 |
| *Methanococcus maripaludis* C6 | NC 009975 | 1769111 | 2 | 0 | 0 | 0 | 4 | 1,29 |
| *Methanococcus maripaludis* C7 | NC 009637 | 1798019 | 3 | 0 | 0 | 0 | 5 | 1,50 |
| *Sulfolobus islandicus* M 14 25 | NC 012588 | 2646102 | 5 | 0 | 0 | 0 | 9 | 1,38 |
| *Sulfolobus islandicus* M 16 4 | NC 012726 | 2623600 | 4 | 0 | 0 | 0 | 9 | 1,52 |
| *Sulfolobus islandicus* Y N 15 51 | NC 012623 | 2852339 | 10 | 0 | 0 | 0 | 17 | 1,55 |
| *Acinetobacter baumannii* AB0057 | NC 011586 | 4108378 | 4 | 0 | 0 | 0 | 10 | 1,49 |
| *Acinetobacter baumannii* ACICU | NC 010611 | 3959890 | 3 | 0 | 0 | 0 | 12 | 1,43 |
| *Acinetobacter baumannii* ATCC 17978 | NC 009085 | 4033558 | 5 | 0 | 0 | 0 | 9 | 1,39 |
| *Acinetobacter baumannii* AYE | NC 010410 | 3992524 | 5 | 0 | 0 | 0 | 10 | 1,35 |
| *Acinetobacter baumannii* SDF | NC 010400 | 3470840 | 10 | 0 | 0 | 0 | 13 | 1,33 |
| *Anaeromyxobacter* K | NC 011145 | 5133942 | 7 | 0 | 0 | 0 | 14 | 1,58 |
| *Bacillus anthracis* A0248 | NC 012659 | 5302097 | 2 | 0 | 0 | 0 | 4 | 1,59 |
| *Bacillus anthracis* Ames | NC 003997 | 5301969 | 4 | 0 | 0 | 0 | 7 | 1,59 |
| *Bacillus anthracis* Ames 0581 | NC 007530 | 5302097 | 4 | 0 | 0 | 0 | 7 | 1,59 |
| *Bacillus anthracis* CDC 684 | NC 012581 | 5304831 | 4 | 0 | 0 | 0 | 6 | 1,53 |
| *Bacillus anthracis* str Sterne | NC 005945 | 5303359 | 5 | 0 | 0 | 0 | 7 | 1,58 |
| *Bacillus cereus* 03BB102 | NC 012472 | 5344909 | 5 | 0 | 0 | 0 | 8 | 1,54 |
| *Bacillus cereus* AH187 | NC 011658 | 5344302 | 3 | 0 | 0 | 0 | 9 | 1,55 |
| *Bacillus cereus* ATCC14579 | NC 004722 | 5489121 | 8 | 0 | 0 | 0 | 12 | 1,53 |
| *Bacillus cereus* ATCC 10987 | NC 003909 | 5298916 | 9 | 0 | 0 | 0 | 11 | 1,44 |
| *Bacillus cereus* B4264 | NC 011725 | 5496451 | 5 | 0 | 0 | 0 | 7 | 1,50 |
| *Bacillus cereus* Q1 | NC 011969 | 5288684 | 9 | 0 | 0 | 0 | 12 | 1,43 |
| *Bacillus cereus* ZK | NC 006274 | 5376643 | 4 | 0 | 0 | 0 | 5 | 1,39 |
| *Bacillus thuringiensis* Al Hakam | NC 008600 | 5332193 | 5 | 0 | 0 | 0 | 6 | 1,54 |
| *Bacillus thuringiensis* konkukian | NC 005957 | 5312507 | 6 | 0 | 0 | 0 | 8 | 1,57 |
| *Bacillus weihenstephanensis* KBAB4 | NC 010184 | 5337958 | 7 | 0 | 0 | 0 | 9 | 1,49 |
| *Bifidobacterium longum* | NC 004307 | 2288878 | 6 | 0 | 0 | 0 | 19 | 1,42 |
| *Bifidobacterium longum* DJO10A | NC 010816 | 2409732 | 7 | 0 | 0 | 0 | 27 | 1,39 |
| *Bordetella parapertussis* | NC 002928 | 4841745 | 3 | 1 | 0 | 0 | 13 | 1,34 |
| *Borrelia afzelii* PKo | NC 008277 | 918329 | 2 | 0 | 0 | 0 | 8 | 1,29 |
| *Borrelia burgdorferi* | NC 001318 | 923735 | 2 | 0 | 0 | 0 | 3 | 1,34 |
| *Borrelia burgdorferi* ZS7 | NC 011728 | 919660 | 2 | 0 | 0 | 0 | 4 | 1,32 |
| *Borrelia garinii* PBi | NC 006156 | 917164 | 2 | 0 | 0 | 0 | 6 | 1,27 |
| *Borrelia turicatae* 91E135 | NC 008710 | 930435 | 2 | 0 | 0 | 0 | 24 | 0,80 |
| *Campylobacter jejuni* RM1221 | NC 003912 | 1803229 | 5 | 0 | 0 | 0 | 5 | 1,33 |
| *Chlamydia muridarum* | NC 002620 | 1088278 | 1 | 0 | 0 | 0 | 1 | 1,10 |
| *Chlamydophila felis* Fe C-56 | NC 007899 | 1182900 | 2 | 1 | 0 | 0 | 4 | 1,10 |
| *Clostridium botulinum* A Hall | NC 009698 | 3814283 | 3 | 0 | 0 | 0 | 17 | 1,69 |
| *Corynebacterium glutamicum* R | NC 009342 | 3361525 | 8 | 0 | 0 | 0 | 19 | 1,33 |
| *Coxiella burnetii* CbuG Q212 | NC 011527 | 2037569 | 3 | 0 | 0 | 0 | 7 | 1,24 |
| *Coxiella burnetii* RSA 331 | NC 010117 | 2045234 | 2 | 0 | 0 | 0 | 6 | 1,16 |
| *Diaphorobacter* TPSY | NC 011992 | 3850810 | 7 | 0 | 0 | 0 | 13 | 1,60 |
| *Escherichia coli* E24377A | NC 009801 | 5050757 | 16 | 0 | 0 | 0 | 33 | 1,23 |
| *Escherichia coli* IAI1 | NC 011741 | 4767711 | 6 | 0 | 0 | 0 | 22 | 1,33 |
| *Escherichia coli* S88 | NC 011742 | 5104158 | 12 | 0 | 0 | 0 | 44 | 1,21 |
| *Escherichia coli* SE11 | NC 011415 | 4957337 | 12 | 0 | 0 | 0 | 36 | 1,22 |
| *Escherichia coli* SMS 3 5 | NC 010498 | 5140795 | 16 | 0 | 0 | 0 | 37 | 1,26 |
| *Escherichia fergusonii* ATCC 35469 | NC 011740 | 4654265 | 5 | 0 | 0 | 0 | 17 | 1,28 |
| *Francisella tularensis holarctica* | NC 007880 | 1923080 | 3 | 0 | 0 | 0 | 9 | 1,02 |
| *Francisella tularensis holarctica* FTNF002 00 | NC 009749 | 1917922 | 4 | 0 | 0 | 0 | 9 | 1,14 |
| *Francisella tularensis holarctica* OSU18 | NC 008369 | 1922809 | 2 | 0 | 0 | 0 | 2 | 1,05 |
| *Francisella tularensis novicida* U112 | NC 008601 | 1937318 | 2 | 0 | 0 | 0 | 4 | 1,26 |
| *Haemophilus influenzae* | NC 000907 | 1856283 | 2 | 0 | 0 | 0 | 3 | 1,13 |
| *Haemophilus influenzae* 86 028NP | NC 007146 | 1941840 | 5 | 0 | 0 | 0 | 6 | 1,34 |
| *Haemophilus influenzae* PittGG | NC 009567 | 1914152 | 3 | 0 | 0 | 0 | 5 | 1,33 |
| *Haemophilus somnus* 129PT | NC 008309 | 2036382 | 5 | 0 | 0 | 0 | 6 | 1,11 |
| *Haemophilus somnus* 2336 | NC 010519 | 2296198 | 10 | 0 | 0 | 0 | 13 | 1,39 |
| *Helicobacter acinonychis Sheeba* | NC 008229 | 1576126 | 3 | 1 | 0 | 0 | 8 | 1,05 |
| *Helicobacter pylori* 26695 | NC 000915 | 1691694 | 2 | 0 | 0 | 0 | 2 | 1,28 |
| *Helicobacter pylori* G27 | NC 011333 | 1676597 | 1 | 0 | 0 | 0 | 1 | 1,49 |
| *Helicobacter pylori* HPAG1 | NC 008086 | 1619172 | 1 | 0 | 0 | 0 | 2 | 1,30 |
| *Helicobacter pylori* J99 | NC 000921 | 1667315 | 1 | 0 | 0 | 0 | 2 | 1,34 |
| *Helicobacter pylori* P12 | NC 011498 | 1697725 | 2 | 0 | 0 | 0 | 2 | 1,27 |
| *Helicobacter pylori* Shi470 | NC 010698 | 1631528 | 1 | 0 | 0 | 0 | 1 | 1,36 |
| *Klebsiella pneumoniae* 342 | NC 011283 | 5721829 | 8 | 0 | 0 | 0 | 26 | 1,30 |
| *Klebsiella pneumoniae* MGH 78578 | NC 009648 | 5391051 | 10 | 0 | 0 | 0 | 26 | 1,23 |
| *Klebsiella pneumoniae* NTUH K2044 | NC 012731 | 5323499 | 4 | 0 | 0 | 0 | 6 | 1,21 |
| *Lactobacillus casei* | NC 010999 | 3123185 | 6 | 0 | 0 | 0 | 15 | 1,12 |
| *Lactobacillus rhamnosus* GG | NC 013198 | 3053113 | 1 | 0 | 0 | 0 | 1 | 1,08 |
| *Legionella pneumophila* Lens | NC 006369 | 3393483 | 6 | 0 | 0 | 0 | 7 | 1,14 |
| *Legionella pneumophila* Paris | NC 006368 | 3553662 | 1 | 0 | 0 | 0 | 4 | 1,19 |
| *Legionella pneumophila* Philadelphia 1 | NC 002942 | 3446294 | 5 | 0 | 0 | 0 | 7 | 1,16 |
| *Listeria monocytogenes* 4b F2365 | NC 002973 | 2946690 | 2 | 0 | 0 | 0 | 3 | 1,15 |
| *Listeria monocytogenes* HCC23 | NC 011660 | 3018730 | 2 | 0 | 0 | 0 | 4 | 1,11 |
| *Listeria welshimeri serovar* 6b SLCC5334 | NC 008555 | 2854332 | 1 | 0 | 0 | 0 | 4 | 1,11 |
| *Mycobacterium avium* 104 | NC 008595 | 5553713 | 11 | 0 | 0 | 0 | 31 | 1,17 |
| *Mycobacterium avium paratuberculosis* | NC 002944 | 4898778 | 7 | 0 | 0 | 0 | 22 | 1,22 |
| *Neisseria gonorrhoeae* FA 1090 | NC 002946 | 2184693 | 3 | 0 | 0 | 0 | 9 | 1,47 |
| *Neisseria gonorrhoeae* NCCP11945 | NC 011035 | 2263912 | 3 | 0 | 0 | 0 | 13 | 1,36 |
| *Neisseria meningitidis* 053442 | NC 010120 | 2184180 | 3 | 0 | 0 | 0 | 7 | 1,54 |
| *Neisseria meningitidis* alpha14 | NC 013016 | 2175943 | 1 | 0 | 0 | 0 | 2 | 1,31 |
| *Neisseria meningitidis* FAM18 | NC 008767 | 2226318 | 2 | 0 | 0 | 0 | 7 | 1,60 |
| *Neisseria meningitidis* MC58 | NC 003112 | 2304823 | 5 | 0 | 0 | 0 | 13 | 1,57 |
| *Neisseria meningitidis* Z2491 | NC 003116 | 2215612 | 2 | 0 | 0 | 0 | 8 | 1,46 |
| *Pectobacterium carotovorum* PC1 | NC 012917 | 4932384 | 1 | 0 | 0 | 0 | 6 | 1,04 |
| *Prochlorococcus marinus* MIT9313 | NC 005071 | 2445315 | 5 | 0 | 0 | 0 | 12 | 1,42 |
| *Prochlorococcus marinus* MIT 9215 | NC 009840 | 1763630 | 1 | 0 | 0 | 0 | 3 | 1,30 |
| *Prochlorococcus marinus* MIT 9312 | NC 007577 | 1733622 | 3 | 0 | 0 | 0 | 4 | 1,19 |
| *Prochlorococcus marinus* MIT 9515 | NC 008817 | 1728522 | 3 | 0 | 0 | 0 | 4 | 1,46 |
| *Prochlorococcus marinus* NATL1A | NC 008819 | 1891371 | 2 | 1 | 0 | 0 | 3 | 1,38 |
| *Prochlorococcus marinus* NATL2A | NC 007335 | 1869227 | 1 | 0 | 0 | 0 | 2 | 1,18 |
| *Pseudomonas aeruginosa* | NC 002516 | 6353896 | 6 | 0 | 0 | 0 | 9 | 1,31 |
| *Pseudomonas aeruginosa* PA7 | NC 009656 | 6682459 | 16 | 0 | 0 | 0 | 26 | 1,40 |
| *Pseudomonas aeruginosa* UCBPP*-*PA14 | NC 008463 | 6631043 | 10 | 0 | 0 | 0 | 19 | 1,31 |
| *Pseudomonas putida* KT2440 | NC 002947 | 6270176 | 18 | 0 | 0 | 0 | 35 | 1,24 |
| *Rhizobium etli* CFN 42 | NC 007761 | 4444203 | 8 | 0 | 0 | 0 | 11 | 1,06 |
| *Rhizobium etli* CIAT 652 | NC 010994 | 4577801 | 5 | 0 | 0 | 0 | 15 | 1,12 |
| *Rhizobium leguminosarum* bv trifolii WSM1325 | NC 012850 | 4835144 | 3 | 0 | 0 | 0 | 3 | 1,00 |
| *Rhizobium leguminosarum* bv trifolii WSM2304 | NC 011369 | 4602776 | 5 | 0 | 0 | 0 | 10 | 1,05 |
| *Rhodopseudomonas palustris* BisB5 | NC 007958 | 4962613 | 8 | 0 | 0 | 0 | 13 | 1,20 |
| *Rhodopseudomonas palustris* TIE 1 | NC 011004 | 5826099 | 6 | 0 | 0 | 0 | 17 | 1,19 |
| *Rickettsia canadensis* McKiel | NC 009879 | 1176341 | 3 | 0 | 0 | 0 | 3 | 0,98 |
| *Rickettsia felis* URRWXCal2 | NC 007109 | 1506365 | 5 | 0 | 0 | 0 | 7 | 1,51 |
| *Salmonella enterica arizonae* serovar 62 z4 z23 | NC 010067 | 4666526 | 9 | 0 | 0 | 0 | 11 | 1,33 |
| *Salmonella enterica* serovar Paratyphi B SPB7 | NC 010102 | 4928300 | 9 | 0 | 0 | 0 | 33 | 1,29 |
| *Salmonella enterica serovar Schwarzengrund CVM19633* | NC 011094 | 4776348 | 8 | 0 | 0 | 0 | 26 | 1,32 |
| *Salmonella enterica* serovar Typhi Ty2 | NC 004631 | 4860418 | 16 | 0 | 0 | 0 | 36 | 1,27 |
| *Salmonella typhi* | NC 003198 | 4877738 | 15 | 0 | 0 | 0 | 36 | 1,31 |
| *Shewanella baltica* OS185 | NC 009665 | 5304396 | 15 | 0 | 0 | 0 | 27 | 1,11 |
| *Shewanella oneidensis* | NC 004347 | 5040801 | 11 | 0 | 0 | 0 | 26 | 1,09 |
| *Sinorhizobium medicae* WSM419 | NC 009636 | 3835932 | 2 | 0 | 0 | 0 | 5 | 1,01 |
| *Staphylococcus aureus aureus* MSSA476 | NC 002953 | 2839800 | 1 | 0 | 0 | 0 | 2 | 1,43 |
| *Staphylococcus aureus* JH1 | NC 009632 | 2948029 | 3 | 0 | 0 | 0 | 8 | 1,64 |
| *Staphylococcus aureus* JH9 | NC 009487 | 2948225 | 3 | 0 | 0 | 0 | 7 | 1,62 |
| *Staphylococcus aureus* Mu3 | NC 009782 | 2921314 | 4 | 0 | 0 | 0 | 8 | 1,42 |
| *Staphylococcus aureus* Mu50 | NC 002758 | 2919651 | 3 | 0 | 0 | 0 | 8 | 1,56 |
| *Staphylococcus aureus* MW2 | NC 003923 | 2860755 | 4 | 0 | 0 | 0 | 4 | 1,33 |
| *Staphylococcus aureus* N315 | NC 002745 | 2855028 | 3 | 0 | 0 | 0 | 10 | 1,49 |
| *Staphylococcus aureus* NCTC 8325 | NC 007795 | 2861667 | 2 | 0 | 0 | 0 | 3 | 1,55 |
| *Staphylococcus aureus* Newman | NC 009641 | 2920025 | 3 | 0 | 0 | 0 | 5 | 1,46 |
| *Staphylococcus aureus* RF122 | NC 007622 | 2781711 | 4 | 0 | 0 | 0 | 5 | 1,47 |
| *Staphylococcus aureus* USA300 FPR3757 | NC 007793 | 2913809 | 4 | 0 | 0 | 0 | 5 | 1,50 |
| *Staphylococcus aureus* USA300 TCH1516 | NC 010079 | 2913957 | 2 | 0 | 0 | 0 | 4 | 1,43 |
| *Streptococcus agalactiae* 2603 | NC 004116 | 2191128 | 4 | 0 | 0 | 0 | 10 | 1,30 |
| *Streptococcus agalactiae* A909 | NC 007432 | 2158237 | 6 | 0 | 0 | 0 | 7 | 1,11 |
| *Streptococcus agalactiae* NEM316 | NC 004368 | 2243078 | 4 | 0 | 0 | 0 | 7 | 1,27 |
| *Streptococcus equi* 4047 | NC 012471 | 2285991 | 6 | 0 | 0 | 0 | 7 | 1,25 |
| *Streptococcus equi zooepidemicus* MGCS10565 | NC 011134 | 2053088 | 3 | 0 | 0 | 0 | 8 | 1,27 |
| *Streptococcus pneumoniae* 70585 | NC 012468 | 2215892 | 5 | 0 | 0 | 0 | 19 | 1,25 |
| *Streptococcus pneumoniae* ATCC 700669 | NC 011900 | 2253049 | 1 | 0 | 0 | 0 | 2 | 1,45 |
| *Streptococcus pneumoniae* CGSP14 | NC 010582 | 2240758 | 4 | 0 | 0 | 0 | 10 | 1,37 |
| *Streptococcus pneumoniae* G54 | NC 011072 | 2108653 | 6 | 0 | 0 | 0 | 21 | 1,37 |
| *Streptococcus pneumoniae* Hungary19A 6 | NC 010380 | 2277696 | 4 | 0 | 0 | 0 | 5 | 1,29 |
| *Streptococcus pneumoniae* JJA | NC 012466 | 2150524 | 5 | 0 | 0 | 0 | 16 | 1,39 |
| *Streptococcus pneumoniae* P1031 | NC 012467 | 2142052 | 5 | 0 | 0 | 0 | 19 | 1,25 |
| *Streptococcus pneumoniae* R6 | NC 003098 | 2067739 | 7 | 0 | 0 | 0 | 18 | 1,43 |
| *Streptococcus pneumoniae* Taiwan19F 14 | NC 012469 | 2142322 | 3 | 0 | 0 | 0 | 19 | 1,43 |
| *Streptococcus pneumoniae* TIGR4 | NC 003028 | 2191712 | 1 | 0 | 0 | 0 | 4 | 1,49 |
| *Streptococcus pyogenes* M1 GAS | NC 002737 | 1878905 | 2 | 0 | 0 | 0 | 4 | 1,30 |
| *Streptococcus pyogenes* Manfredo | NC 009332 | 1867575 | 1 | 0 | 0 | 0 | 3 | 1,36 |
| *Streptococcus pyogenes* MGAS10270 | NC 008022 | 1955799 | 4 | 0 | 0 | 0 | 6 | 1,36 |
| *Streptococcus pyogenes* MGAS10394 | NC 006086 | 1927019 | 4 | 0 | 0 | 0 | 6 | 1,40 |
| *Streptococcus pyogenes* MGAS10750 | NC 008024 | 1964785 | 2 | 0 | 0 | 0 | 5 | 1,24 |
| *Streptococcus pyogenes* MGAS2096 | NC 008023 | 1886932 | 2 | 0 | 0 | 0 | 4 | 1,21 |
| *Streptococcus pyogenes* MGAS315 | NC 004070 | 1927672 | 2 | 0 | 0 | 0 | 3 | 1,27 |
| *Streptococcus pyogenes* MGAS5005 | NC 007297 | 1864820 | 4 | 0 | 0 | 0 | 6 | 1,27 |
| *Streptococcus pyogenes* MGAS6180 | NC 007296 | 1924682 | 5 | 0 | 0 | 0 | 7 | 1,48 |
| *Streptococcus pyogenes* MGAS8232 | NC 003485 | 1922089 | 4 | 0 | 0 | 0 | 7 | 1,19 |
| *Streptococcus pyogenes* MGAS9429 | NC 008021 | 1862703 | 2 | 0 | 0 | 0 | 6 | 1,25 |
| *Streptococcus pyogenes* NZ131 | NC 011375 | 1841725 | 3 | 0 | 0 | 0 | 4 | 1,50 |
| *Thermotoga neapolitana* DSM 4359 | NC 011978 | 1911485 | 4 | 0 | 0 | 0 | 5 | 1,11 |
| *Thermotoga petrophila* RKU-1 | NC 009486 | 1849562 | 1 | 0 | 0 | 0 | 3 | 1,07 |
| *Wolbachia* endosymbiont of Drosophila melanogaster | NC 002978 | 1285894 | 4 | 0 | 0 | 0 | 5 | 1,09 |
| *Wolbachia* wRi | NC 012416 | 1466529 | 6 | 0 | 0 | 0 | 7 | 1,29 |
| *Xanthomonas oryzae* PXO99A | NC 010717 | 5314934 | 16 | 0 | 0 | 0 | 30 | 1,49 |
| *Xylella fastidiosa* M12 | NC 010513 | 2510489 | 7 | 0 | 0 | 0 | 20 | 1,62 |
| *Yersinia pestis* KIM 10 | NC 004088 | 4666481 | 10 | 0 | 0 | 0 | 22 | 1,25 |
| *Yersinia pseudotuberculosis* IP 31758 | NC 009708 | 4790782 | 10 | 0 | 0 | 0 | 21 | 1,20 |
| *Yersinia pseudotuberculosis* YPIII | NC 010465 | 4756434 | 13 | 0 | 0 | 0 | 26 | 1,17 |
| **Total** |  |  | **1787** | **11** | **134** | **271** | **3847** |  |
