# Supplementary material for: A quantitative account of genomic island acquisitions in prokaryotes
Source: BMC Genomics. 2011 Aug 24;12:427. doi: 10.1186/1471-2164-12-427 (PMC3176501; doi:10.1186/1471-2164-12-427)
Supplement: Additional file 3 — List of genomes with clustered GIs. List of genomes with clusters in which more than 1 GI are located, using the compositional threshold of CI-10. [file 1471-2164-12-427-S3.DOCX]

Supplementary File 3. List of genomes with clusters in which more than 1 GI are located, using the compositional threshold of CI-10.

| **Organism** | **Accession** | **Nr of basepairs** | **Nr of GIs longer than threshold** | **Nr of GIs with d* with Genome < cutoff** | **Nr of clusters under cutoff** | **Total nr of GIs in clusters under cutoff** | **Total length of GIs in clusters under cutoff** | **Total nr of GIs** | **Dissimilarity between genome and CI-10** | **Percentage of the genome taken up by these GI clusters** |
| --- | --- | --- | --- | --- | --- | --- | --- | --- | --- | --- |
| *Escherichia coli* O157H7 | NC 002695 | 5577000 | 24 | 0 | 7 | 15 | 124119 | 59 | 1,44 | 2,23 |
| *Mycobacterium marinum* M | NC 010612 | 6731639 | 21 | 1 | 4 | 8 | 65185 | 24 | 1,38 | 0,97 |
| *Shewanella baltica* OS155 | NC 009052 | 5200625 | 20 | 0 | 4 | 8 | 44550 | 30 | 1,11 | 0,86 |
| *Escherichia coli* C ATCC 8739 | NC 010468 | 4814022 | 15 | 0 | 3 | 6 | 55773 | 27 | 1,26 | 1,16 |
| *Escherichia coli* K 12 substr W3110 | AC 000091 | 4712709 | 17 | 0 | 3 | 6 | 53391 | 32 | 1,22 | 1,13 |
| *Mycobacterium ulcerans* Agy99 | NC 008611 | 5712058 | 17 | 0 | 3 | 6 | 68885 | 29 | 1,09 | 1,21 |
| *Pseudomonas putida* F1 | NC 009512 | 6045107 | 17 | 0 | 3 | 6 | 47013 | 29 | 1,29 | 0,78 |
| *Pseudomonas putida* GB 1 | NC 010322 | 6165265 | 17 | 0 | 3 | 6 | 65144 | 23 | 1,40 | 1,06 |
| *Rhodobacter sphaeroides* ATCC 17025 | NC 009428 | 3263694 | 13 | 0 | 3 | 7 | 95690 | 16 | 1,26 | 2,93 |
| *Shewanella* W3-18-1 | NC 008750 | 4775643 | 20 | 1 | 3 | 6 | 71481 | 26 | 1,09 | 1,50 |
| *Streptococcus pyogenes* SSI-1 | NC 004606 | 1921337 | 10 | 2 | 3 | 6 | 43775 | 13 | 1,24 | 2,28 |
| *Xanthomonas oryzae* KACC10331 | NC 006834 | 5012031 | 25 | 0 | 3 | 6 | 65366 | 31 | 1,38 | 1,30 |
| *Bifidobacterium longum* infantis ATCC 15697 | NC 011593 | 2873216 | 15 | 0 | 2 | 4 | 26243 | 42 | 1,41 | 0,91 |
| Bradyrhizobium ORS278 | NC 009445 | 7563110 | 18 | 2 | 2 | 5 | 29080 | 36 | 1,15 | 0,38 |
| *Clostridium botulinum* A2 Kyoto | NC 012563 | 4214640 | 7 | 0 | 2 | 4 | 33235 | 21 | 1,86 | 0,79 |
| *Clostridium botulinum* F Langeland | NC 009699 | 4052464 | 5 | 0 | 2 | 4 | 23150 | 20 | 1,81 | 0,57 |
| *Escherichia coli APEC O1* | NC 008563 | 5154626 | 14 | 0 | 2 | 4 | 44690 | 51 | 1,30 | 0,87 |
| *Escherichia coli* O157 H7 EC4115 | NC 011353 | 5651677 | 22 | 0 | 2 | 4 | 44786 | 56 | 1,41 | 0,79 |
| *Methylobacterium extorquens* PA1 | NC 010172 | 5549314 | 9 | 0 | 2 | 4 | 35389 | 12 | 1,56 | 0,64 |
| *Salmonella enterica* Choleraesuis | NC 006905 | 4823639 | 13 | 0 | 2 | 4 | 35091 | 40 | 1,21 | 0,73 |
| *Salmonella enterica* serovar Agona SL483 | NC 011149 | 4867213 | 10 | 0 | 2 | 4 | 26077 | 30 | 1,23 | 0,54 |
| *Salmonella enterica* serovar Dublin CT 02021853 | NC 011205 | 4912093 | 11 | 0 | 2 | 4 | 24062 | 27 | 1,33 | 0,49 |
| *Shewanella baltica* OS223 | NC 011663 | 5219415 | 11 | 0 | 2 | 4 | 38227 | 24 | 1,10 | 0,73 |
| *Shewanella halifaxensis* HAW EB4 | NC 010334 | 5301588 | 9 | 0 | 2 | 4 | 34147 | 19 | 1,32 | 0,64 |
| *Shigella boydii* CDC 3083 94 | NC 010658 | 4681940 | 18 | 0 | 2 | 4 | 59843 | 44 | 1,22 | 1,28 |
| *Stenotrophomonas maltophilia* K279a | NC 010943 | 4920428 | 11 | 0 | 2 | 4 | 23295 | 26 | 1,47 | 0,47 |
| *Sulfolobus islandicus* L S 2 15 | NC 012589 | 2775362 | 8 | 0 | 2 | 4 | 29230 | 11 | 1,55 | 1,05 |
| *Xanthomonas oryzae* MAFF 311018 | NC 007705 | 5010792 | 27 | 0 | 2 | 4 | 37957 | 38 | 1,37 | 0,76 |
| *Xylella fastidiosa* | NC 002488 | 2717582 | 12 | 0 | 2 | 4 | 21335 | 20 | 1,70 | 0,79 |
| *Yersinia pseudotuberculosis* IP32953 | NC 006155 | 4812453 | 13 | 0 | 2 | 4 | 41374 | 24 | 1,24 | 0,86 |
| *Anaeromyxobacter dehalogenans* 2CP-C | NC 007760 | 5085101 | 7 | 0 | 1 | 2 | 45647 | 20 | 1,43 | 0,90 |
| *Bacillus cereus* AH820 | NC 011773 | 5378436 | 6 | 0 | 1 | 2 | 12021 | 10 | 1,48 | 0,22 |
| *Bacillus cereus* G9842 | NC 011772 | 5464296 | 7 | 0 | 1 | 2 | 14741 | 13 | 1,48 | 0,27 |
| *Bordetella bronchiseptica* | NC 002927 | 5415453 | 9 | 0 | 1 | 2 | 10137 | 24 | 1,35 | 0,19 |
| *Bordetella pertussis* | NC 002929 | 4144564 | 13 | 0 | 1 | 2 | 12174 | 23 | 1,25 | 0,29 |
| *Clostridium botulinum* A | NC 009495 | 3942444 | 3 | 0 | 1 | 2 | 18305 | 17 | 1,75 | 0,46 |
| *Clostridium botulinum* A3 Loch Maree | NC 010520 | 4049948 | 5 | 0 | 1 | 2 | 17839 | 22 | 1,83 | 0,44 |
| *Clostridium botulinum* A ATCC 19397 | NC 009697 | 3918643 | 3 | 0 | 1 | 2 | 17946 | 16 | 1,65 | 0,46 |
| *Clostridium botulinum* B1 Okra | NC 010516 | 4014780 | 8 | 0 | 1 | 2 | 25765 | 20 | 1,78 | 0,64 |
| *Clostridium botulinum* Ba4 657 | NC 012658 | 4034620 | 6 | 0 | 1 | 2 | 36548 | 21 | 1,95 | 0,91 |
| *Escherichia coli* 536 | NC 008253 | 5009476 | 12 | 0 | 1 | 2 | 13794 | 37 | 1,26 | 0,28 |
| *Escherichia coli* HS | NC 009800 | 4709875 | 12 | 0 | 1 | 2 | 31457 | 27 | 1,27 | 0,67 |
| *Escherichia coli* IAI39 | NC 011750 | 5205384 | 21 | 0 | 1 | 2 | 37162 | 49 | 1,30 | 0,71 |
| *Escherichia coli* K 12 substr MG1655 | NC 000913 | 4705957 | 16 | 0 | 1 | 2 | 48013 | 32 | 1,18 | 1,02 |
| *Escherichia coli* UMN026 | NC 011751 | 5276406 | 14 | 0 | 1 | 2 | 27192 | 37 | 1,29 | 0,52 |
| *Escherichia coli* UTI89 | NC 007946 | 5138109 | 11 | 0 | 1 | 2 | 15234 | 46 | 1,29 | 0,30 |
| *Lactobacillus casei* ATCC 334 | NC 008526 | 2936625 | 9 | 0 | 1 | 2 | 34463 | 14 | 1,15 | 1,17 |
| *Legionella pneumophila* Corby | NC 009494 | 3627563 | 7 | 0 | 1 | 2 | 17369 | 9 | 1,11 | 0,48 |
| *Listeria innocua* | NC 003212 | 3054226 | 4 | 0 | 1 | 2 | 34205 | 5 | 1,31 | 1,12 |
| *Listeria monocytogenes* Clip81459 | NC 012488 | 2954300 | 3 | 0 | 1 | 2 | 15460 | 4 | 1,27 | 0,52 |
| *Methanococcus maripaludis* S2 | NC 005791 | 1684868 | 2 | 0 | 1 | 2 | 16757 | 2 | 1,32 | 0,99 |
| *Methylobacterium extorquens* AM1 | NC 012808 | 5590056 | 8 | 0 | 1 | 2 | 22486 | 14 | 1,40 | 0,40 |
| *Methylobacterium extorquens* DM4 | NC 012988 | 6028679 | 7 | 1 | 1 | 2 | 28191 | 10 | 1,54 | 0,47 |
| *Methylobacterium populi* BJ001 | NC 010725 | 5883305 | 14 | 0 | 1 | 2 | 11107 | 17 | 1,35 | 0,19 |
| *Pseudomonas syringae* pv B728a | NC 007005 | 6180751 | 15 | 0 | 1 | 2 | 15870 | 37 | 1,20 | 0,26 |
| *Rhizobium leguminosarum* bv viciae 3841 | NC 008380 | 5129387 | 8 | 0 | 1 | 2 | 36242 | 16 | 1,28 | 0,71 |
| *Rickettsia massiliae* MTU5 | NC 009900 | 1380340 | 4 | 0 | 1 | 2 | 33328 | 4 | 1,32 | 2,41 |
| *Salmonella enterica* Paratypi ATCC 9150 | NC 006511 | 4650733 | 10 | 0 | 1 | 2 | 13495 | 27 | 1,30 | 0,29 |
| *Salmonella enterica* serovar Enteritidis P125109 | NC 011294 | 4752789 | 9 | 0 | 1 | 2 | 14847 | 28 | 1,20 | 0,31 |
| *Salmonella enterica* serovar Gallinarum 287 91 | NC 011274 | 4725250 | 9 | 0 | 1 | 2 | 25489 | 25 | 1,17 | 0,54 |
| *Salmonella enterica* serovar Newport SL254 | NC 011080 | 4896608 | 8 | 0 | 1 | 2 | 26586 | 28 | 1,29 | 0,54 |
| *Salmonella enterica* serovar Paratyphi A AKU 12601 | NC 011147 | 4647252 | 10 | 0 | 1 | 2 | 13510 | 27 | 1,28 | 0,29 |
| *Salmonella enterica* serovar Paratyphi C RKS4594 | NC 012125 | 4902124 | 9 | 0 | 1 | 2 | 10922 | 32 | 1,22 | 0,22 |
| *Salmonella typhimurium* LT2 | NC 003197 | 4926824 | 14 | 0 | 1 | 2 | 11305 | 35 | 1,23 | 0,23 |
| *Shewanella* ANA-3 | NC 008577 | 5043236 | 8 | 0 | 1 | 2 | 24701 | 14 | 1,30 | 0,49 |
| *Shewanella baltica* OS195 | NC 009997 | 5423673 | 14 | 0 | 1 | 2 | 41460 | 33 | 1,08 | 0,76 |
| *Shewanella* MR-4 | NC 008321 | 4773520 | 9 | 0 | 1 | 2 | 22512 | 15 | 1,21 | 0,47 |
| *Shewanella* MR-7 | NC 008322 | 4861076 | 8 | 0 | 1 | 2 | 11847 | 18 | 1,31 | 0,24 |
| *Shewanella pealeana* ATCC 700345 | NC 009901 | 5248504 | 10 | 0 | 1 | 2 | 10500 | 21 | 1,21 | 0,20 |
| *Shewanella putrefaciens* CN-32 | NC 009438 | 4725781 | 15 | 0 | 1 | 2 | 10250 | 27 | 1,16 | 0,22 |
| *Shigella boydii* Sb227 | NC 007613 | 4584392 | 19 | 0 | 1 | 2 | 17149 | 39 | 1,21 | 0,37 |
| *Shigella* flexneri 2a 2457T | NC 004741 | 4665060 | 20 | 0 | 1 | 2 | 10692 | 41 | 1,28 | 0,23 |
| *Sinorhizobium meliloti* | NC 003047 | 3706337 | 6 | 0 | 1 | 2 | 13498 | 9 | 1,11 | 0,36 |
| *Staphylococcus aureus aureus* MRSA252 | NC 002952 | 2944085 | 8 | 0 | 1 | 2 | 10318 | 12 | 1,50 | 0,35 |
| *Stenotrophomonas maltophilia* R551 3 | NC 011071 | 4639312 | 6 | 0 | 1 | 2 | 13149 | 19 | 1,32 | 0,28 |
| *Streptococcus equi zooepidemicus* | NC 012470 | 2180581 | 7 | 0 | 1 | 2 | 10052 | 11 | 1,33 | 0,46 |
| *Streptococcus pneumoniae* D39 | NC 008533 | 2075346 | 6 | 0 | 1 | 2 | 12846 | 14 | 1,46 | 0,62 |
| *Sulfolobus islandicus* M 16 27 | NC 012632 | 2730865 | 5 | 0 | 1 | 2 | 11053 | 8 | 1,48 | 0,40 |
| *Sulfolobus islandicus* Y G 57 14 | NC 012622 | 2740659 | 4 | 0 | 1 | 2 | 10449 | 12 | 1,51 | 0,38 |
| *Xanthomonas campestris* 8004 | NC 007086 | 5222261 | 14 | 0 | 1 | 2 | 60879 | 31 | 1,56 | 1,17 |
| *Xanthomonas campestris* ATCC 33913 | NC 003902 | 5148705 | 15 | 0 | 1 | 2 | 12046 | 32 | 1,54 | 0,23 |
| *Xanthomonas campestris* B100 | NC 010688 | 5151560 | 11 | 0 | 1 | 2 | 12630 | 23 | 1,49 | 0,25 |
| *Xanthomonas campestris vesicatoria* 85-10 | NC 007508 | 5252445 | 15 | 0 | 1 | 2 | 10421 | 34 | 1,54 | 0,20 |
| *Xanthomonas citri* | NC 003919 | 5249491 | 13 | 0 | 1 | 2 | 11500 | 27 | 1,51 | 0,22 |
| *Yersinia pestis* Angola | NC 010159 | 4568601 | 15 | 0 | 1 | 2 | 19035 | 27 | 1,04 | 0,42 |
| *Yersinia pseudotuberculosis* PB1 | NC 010634 | 4762700 | 10 | 0 | 1 | 2 | 20550 | 19 | 1,17 | 0,43 |
|  |  |  |  |  |  |  |  |  |  |  |
| **Total** |  |  | **990** | **7** | **134** | **271** |  |  |  |  |
