# Supplementary material for: A quantitative account of genomic island acquisitions in prokaryotes
Source: BMC Genomics. 2011 Aug 24;12:427. doi: 10.1186/1471-2164-12-427 (PMC3176501; doi:10.1186/1471-2164-12-427)
Supplement: Additional file 4 — Clustered GIs in multichromosomal genomes. Clusters of GIs in genomes with multiple chromosomes using cut-off of CI-10. Highlighted in green are clustered GIs that are located on a different replicon. [file 1471-2164-12-427-S4.DOCX]

Supplementary File 4. Clusters of GIs in genomes with multiple chromosomes using cut-off of CI-10. Highlighted in green are clustered GIs that are located on a different replicon.

| **Organism** | **Accession number (largest chromosome)** | **Nr of GIs longer than threshold** | **Nr of clusters under cutoff** | **Total nr of GIs in clusters under cutoff** | **Cutoff (CI-10)** | **Info on clusters under cutoff (nr of GIs, total length of GIs, cluster)** | **Info on clusters under cutoff (nr of GIs, total length of GIs, cluster)** | **Info on clusters under cutoff (nr of GIs, total length of GIs, cluster)** |
| --- | --- | --- | --- | --- | --- | --- | --- | --- |
| *Burkholderia* 383 | NC 007510 | 5 | 0 | 0 | 1,34 |  |  |  |
| *Brucella* *suis* ATCC 23445 | NC 010169 | 6 | 0 | 0 | 1,05 |  |  |  |
| *Vibrio parahaemolyticus* | NC 004603 | 11 | 0 | 0 | 1,25 |  |  |  |
|  |  |  |  |  |  |  |  |  |
| *Burkholderia cenocepacia* J2315 | NC 011000 | 0 | 1 | 2 | 1,60 | (NC 011001 GI12:1.46399012,NC 011001 GI14:1.46399012) |  |  |
| ***Burkholderia mallei* NCTC 10229** | NC 008836 | 6 | 1 | 2 | 1,44 | (NC 008835 GI03:1.21183769,NC 008836 GI06:1.21183769) |  |  |
| *Burkholderia phytofirmans* PsJN | NC 010681 | 6 | 1 | 2 | 1,36 | (NC 010681 GI03:0.4978436655,NC 010681 GI09:0.4978436655) |  |  |
| *Burkholderia pseudomallei* 1106a | NC 009076 | 10 | 1 | 2 | 1,69 | (NC 009076 GI14:1.455098458,NC 009076 GI15:1.455098458) |  |  |
| *Ralstonia pickettii* 12D | NC 012856 | 4 | 1 | 2 | 1,34 | (NC 012856 GI01:1.280336597,NC 012856 GI02:1.280336597) |  |  |
| ***Burkholderia mallei* ATCC 23344** | NC 006348 | 10 | 2 | 4 | 1,49 | (NC 006348 GI11:1.46568634,NC 006349 GI07:1.46568634) | (NC 006348 GI06:0.482204303,NC 006349 GI14:0.482204303) |  |
| ***Burkholderia mallei* SAVP1** | NC 008785 | 9 | 2 | 4 | 1,73 | (NC 008785 GI05:1.140204304,NC 008785 GI17:1.140204304) | (NC 008784 GI10:1.639058802,NC 008785 GI14:1.639058802) |  |
| *Burkholderia vietnamiensis* G4 | NC 009256 | 13 | 2 | 4 | 1,84 | (NC 009256 GI08:1.199337892,NC 009256 GI17:1.199337892) | (NC 009256 GI14:1.666500227,NC 009256 GI15:1.666500227) |  |
| *Vibrio vulnificus* CMCP6 | NC 004459 | 10 | 2 | 4 | 1,30 | (NC 004459 GI07:1.004154256,NC 004459 GI08:1.004154256) | (NC 004459 GI01:1.211210003,NC 004459 GI06:1.211210003) |  |
| *Burkholderia mallei* NCTC 10247 | NC 009080 | 9 | 3 | 6 | 1,55 | (NC 009080 GI09:1.237163186,NC 009080 GI16:1.237163186) | (NC 009079 CI-25:1.483578558,(NC 009080 GI09:1.237163186,NC 009080 GI16:1.237163186):0.2464153715) | (NC 009080 GI03:1.094577517,NC 009080 GI15:1.094577517) |
| ***Burkholderia pseudomallei* K96243** | NC 006350 | 11 | 3 | 6 | 1,66 | (NC 006350 GI10:1.64998101,NC 006351 GI08:1.64998101) | (NC 006350 GI12:1.575591017,NC 006350 GI13:1.575591017) | (NC 006350 GI05:1.515065379,NC 006350 GI06:1.515065379) |
| **Total** |  | **110** | **19** | **38** |  |  |  |  |
